# The single-cell protein from *Candida utilis* as a functional protein ingredient for broilers

**DOI:** 10.1016/j.psj.2026.107749

**Published:** 2026-09-11

**Authors:** S. Tan, J.S. Ma, X.Y. Su, X.X. Xu, H.Q. Huang, T. Tu, F.F. Guan, H.Y. Luo, H.M. Yu, B. Yao, Y. Wang

**Affiliations:** a State Key Laboratory of Animal Nutrition and Feeding, Institute of Animal Sciences, Chinese Academy of Agricultural Sciences, Beijing, 10093, China; b College of Animal Science and Technology, Northwest A&F University, Yangling, Shaanxi, China; c Biotecnology Research Institute, Chinese Academy of Agricultural Sciences, Beijing, 10081, China

**Keywords:** *Candida utilis* protein, Brolier, Growth, Digestibility, Microbiota and mycobiota

## Abstract

The search for sustainable protein alternatives to soybean meal (SBM) in broilers diets has highlighted single-cell proteins as a promising candidate. *Candida utilis* protein (CUP) represents a potential substitute, yet its optimal inclusion level in broiler nutrition remains to be determined. A 42-day trial was conducted with 512 Arbor Acres broilers (1d old, initial body weights 45.00 ± 0.13 g) were purchased and randomly assigned to eight treatment groups. Treatment diets included 0% control group (CON), 1% CUP group (CUP1), 2% CUP group (CUP2), 3% CUP group (CUP3), 4% CUP group (CUP4), 5% CUP group (CUP5), 6% CUP group (CUP6) and 8% CUP group (CUP8). Growth performance exhibited a quadratic response (*P* < 0.001), with every phase average daily gain and average daily feed intake peaking at 4% inclusion and declining at 8%. Apparent digestibility of dry matter, crude protein and total amino acids was highest at the 3-4% inclusion levels (*P* < 0.05). Serum immunoglobulins immunoglobulin-Y (IgY), immunoglobulin-A (IgA), immunoglobulin-M (IgM), anti-inflammatory cytokines interleukin-4 (IL-4), interleukin-10 (IL-10) and antioxidant enzyme activities superoxide dismutase (SOD), glutathione peroxidase (GSH-Px), total antioxidant capacity (T-AOC) increased linearly with yeast supplementation (*P* < 0.001), whereas pro-inflammatory cytokines interleukin-2 (IL-2), tumor necrosis factor α (TNF-α), interferon gamma (IFN-γ), C-reactive protein (CRP) and malondialdehyde (MDA) decreased linearly (*P* < 0.001). Spleen weight and abdominal fat percentage also rose with increasing inclusion. Microbial community analysis revealed numerical reductions in α diversity with increasing dietary CUP inclusion, whereas principal coordinate analysis demonstrated clear separation of bacterial communities across treatments, with the % CUP group showing the most pronounced deviation from the control group. These results demonstrate quadratic regression analysis using growth performance as the core indicator revealed a theoretically optimal CUP supplementation range of 1.6% to 2.5%. Combined with the measured application effects, the comprehensive recommended optimal inclusion level of CUP in broiler diets is 1.6% to 4.0%, with a recommended level of 4% for large-scale commercial production.

## Introduction

Soybean meal (SBM) is a widely used and highly suitable protein source in broiler diets, characterized by high protein and energy contents, low crude fiber level, and a well-balanced amino acid (AA) profile that closely aligns with broiler nutritional requirements (CVB, 2021). However, the heavy reliance of China and many other countries on importing large quantities of SBM from South America has raised significant concerns regarding the environmental sustainability and supply security of birds meat production (Wangui et al., 2025; Wang et al., 2025). This dependence underscores the critical need to identify and validate sustainable alternative protein sources for the poultry industry. Single-cell proteins derived from microorganisms represent a promising solution. *Candida utilis* (syn. *Cyberlindnera jadinii*), in particular, stands out as a candidate with strong sustainability attributes. Its production requires minimal land input, features rapid cultivation cycles, and can utilize various renewable feedstocks, including agro-industrial by-products and wastes, thereby contributing to a sustainable bioeconomy (Salazar-López et al., 2022). For instance, studies have demonstrated the successful use of orange peel residues as a carbon source for *C. utilis* cultivation, yielding biomass with a high crude protein content and substantiating the efficacy of bioconversion strategies for agricultural residues (Carranza-Méndez et al., 2022; Kurcz et al., 2018). *C. utilis* is a well-recognized GRAS (Generally Recognized as Safe) organism with a long history of safe application in animal feed (Buerth et al., 2016). Nutritionally, *C. utilis* biomass possesses a high crude protein (38-56% on a dry matter basis, Cruz et al., 2020) and a favorable amino acid profile, with particularly high levels of lysine and threonine (Øverland et al., 2013). In terms of cultivation characteristics, *C. utilis* is a yeast species with broad temperature tolerance, acid resistance, and strong robustness under stress conditions (Buerth et al., 2016; Sousa-Silva et al., 2021). In addition to its fundamental nutrient contribution *C. utilis* biomass contains bioactive cell wall components, such as β-glucans and mannans, which have been shown to enhance gut health and immune function in broilers (Kyoung et al., 2023). The application potential of *C. utilis* biomass is further supported by its successful use as a partial replacement for conventional protein sources (e.g., fishmeal and SBM) in aquaculture and swine diets, with no adverse effects on growth performance and even demonstrated benefits for intestinal health (Øverland et al., 2013; Cruz et al., 2019; Grammes et al., 2019). To date, only two peer-reviewed studies have investigated the effects of dietary *Candida utilis* protein (CUP) supplementation in broiler chickens. One study demonstrated that dietary CUP inclusion at levels up to 10% exerted no adverse effects on growth performance (Cruz et al., 2020). In contrast, the second study, which drew its conclusions from alterations in growth performance and plasma metabolome profiles, proposed a potential upper inclusion limit of 4% CUP in broiler diets (Dayan et al., 2026).

Despite this promising background, a systematic, dose-response evaluation of CUP as an alternative to SBM across graded inclusion levels there is currently a paucity of data regarding its application in broiler formulations. Such an assessment is necessary to define not only its effects on growth performance but also its impact on nutrient digestibility, systemic immune and antioxidant status, and gut microbiota-especially at higher inclusion levels where bioactive cell wall components may exert more pronounced effects.

Therefore, this study comprehensively investigated the effects of dietary CUP supplementation at 0-8% inclusion levels on growth performance, nutrient digestibility, immune function, antioxidant status, and cecal microbiota and metabolites in broiler chickens.

## Materials and methods

### Animal ethics statement

The experimental protocol was reviewed and approved by the Laboratory Animal Welfare and Ethics Committee of the Institute of Animal Sciences, Chinese Academy of Agricultural Sciences. The ethics approval code is: IAS2025-221.

### Nutrient composition and amino acid content of CUP

The *C. utilis* strain (CGMCC 2.615) was provided by the China General Microbiological Culture Collection Center. This thermotolerant feed-grade yeast was used to produce CUP via submerged fermentation. The fermentation medium consisted of 2% glucose, 1% peptone, 0.5% yeast extract and 1% corn steep liquor, prepared on the basis of 1 ton of water. Medium pH was adjusted to 5.0-6.0, and sterilization was performed at 121°C for 15-20 min. Fermentation was conducted in a bioreactor with an inoculum size of 5% (v/v). The process lasted 60 h at 37°C, with an agitation speed of 200 rpm and an aeration rate of 1:1.5 (v/v/v). Fermentation was terminated when wet cell weight reached 300 g/L. The collected biomass was dried by centrifugal spray drying under the following conditions: inlet air temperature 160°C, outlet air temperature 80°C, and centrifugal speed 4000 rpm. The chemical composition of the final CUP product is listed in Table 1.

**Table 1:** The chemical composition and non-starch polysaccharides level of *Candida utilis* protein and Soybean meal (%, as is basis)^a^.

Item	*Candida utilis* protein^a^	Soybean meal^b^
**Chemical composition**		
Dry matter	91.0	89.0
Crude protein	38.3	44.3
Ether extract	6.67	1.50
Gross energy, MJ/kg	19.0	17.5
Phosphorus	1.62	0.330
Calcium	0.040	0.780
**Essential amino acids**		
Arginine	1.11	3.38
Histidine	0.360	1.17
Isoleucine	1.35	1.99
Leucine	2.08	3.35
Lysine	2.08	2.68
Phenylalanine	1.28	2.21
Methionine	0.420	0.590
Threonine	1.61	1.71
Valine	1.14	2.09
**Nonessential amino acids**		
Cystine	0.410	0.710
Tyrosine	1.24	1.61
Aspartic	5.19	5.90
Serine	1.80	2.48
Glutamic	6.86	8.65
Glycine	1.30	2.17
Alanine	2.28	2.05
Proline	1.36	2.39
**Non-starch polysaccharides**		
β-glucan	23.6	-
Mannan	5.10	-

a All item are analyzed values.

b All data are from the China Feed Database- Tables of Feed Composition and Nutritive Values in China (in Chinese) 2020.

The price of the CUP product used in experiment was 373 USD per ton (38% crude protein CUP, 9.82 USD per 1% CP). The market price of SBM on the reference date (462 USD per ton, 10.74 USD per 1% CP) was adopted as the baseline, and the CUP price was adjusted in line with changes in soybean meal price during the calculation.

Dry matter (DM) was determined following AOAC (2006) methods 930.15 and 925.10. Nitrogen content was measured by the Kjeldahl method AOAC (2006) using a Kjeldahl nitrogen analyzer (Model KDY-9820, Shandong Haineng Scientific Instruments Co., Ltd., Dezhou, China); Crude protein (CP) was calculated as nitrogen × 6.25; Ether extract (EE) was measured according to AOAC (2006) method 954.02 using an automatic fat analyzer (Model SOX-406, Shandong Haineng Scientific Instruments Co., Ltd.). The gross energy (GE) was measured according to the method of ISO 9831:1998 using a Parr 6400 automatic adiabatic calorimeter (Parr instrument company, Moline, IL). Phosphorus and Cal-ciumwere analyzed as per the method ISO17294-2: 2023. Amino acid composition of feed and fecal samples was determined per AOAC (2006) method 994.12 using an amino acid analyzer (Biochrom 30, UK). Amino acids were derivatized post-column with ninhydrin and quantified by absorbance at 570 nm and 440 nm. Sixteen amino acids were quantified based on chromatographic peak areas using OPENLAB software. Titanium dioxide (TiO₂, 0.2%) was used as an indigestible marker, TiO₂ content in feed and feces was determined according to the methods of (Short et al., 1996). The contents of β-glucans and mannans in CUP were determined according to the method described by NY/T 3477-2019 (2019). The change in reducing sugar content following the reaction of cell wall β-glucan and mannan with β-1,3-glucanase, cellulose (β-1,4-glucanase), β-1,3-1,4-glucanase and β-mannanase, respectively, was determined according to the method described by (Qi et al., 2004).

### Broiler husbandry, experimental design, and dietary treatments

A total of 512 one-day-old male Arbor Acres (AA) broilers (initial body weights 45.00 ± 0.13 g) were purchased and randomly assigned to eight treatment groups. All broiler chicks were placed in a 3-tier-cage stainless steel barrier with one-side trough feeders. 8 replicates were allocated to each of the control and the CUP inclusion groups, for a total of 64 pens with 8 birds per pen. During the first 3 days, house temperature was maintained at 33°C, then reduced by 1°C every 2 days until reaching 25°C. Relative humidity was kept at 50-60 %. Feed and water were available ad libitum throughout the experiment. Lighting was provided 24 h for the first week after hatching, and, thereafter, a light pattern of 23 h light:1 h dark was adopted for the other experimental period.

The trial consisted of two phases: starter phase (days 1-21) and grower phase (days 22-42). Isonitrogenous diets were formulated for each feeding phase in accordance with the Chinese agricultural standard NY/T 33-2004 *Nutritional Requirements of Chickens*. All diets met or exceeded the full nutrient requirements of broilers, with the starter diet (d 1-21) provided in crumbled pellet form and the grower diet (d 22-42) in pelleted form. Within each phase, diets had identical levels of calcium, phosphorus, methionine, lysine, and threonine; the only variable was the inclusion level of CUP. TiO₂ was added as an indigestible marker for digestibility determinations. The experimental diets were formulated to contain graded levels of CUP at inclusion rates of 0% control group (CON), 1% CUP group (CUP1), 2% CUP group (CUP2), 3% CUP group (CUP3), 4% CUP group (CUP4), 5% CUP group (CUP5), 6% CUP group (CUP6) and 8% CUP group (CUP8), and all broilers were offered crumbled feed for the first 7 days, thereafter, pelleted diets were provided for the duration of the trial. The detailed ingredient composition and calculated nutrient contents of experimental diets are summarized in Table 2.

**Table 2:** Calculated and analyzed values of the diets in the starter phase and grower phase (g/kg, as is basis)^a^.

Item	Starter diets								Grower diets							
	CON	CUP1	CUP2	CUP3	CUP4	CUP5	CUP6	CUP8	CON	CUP1	CUP2	CUP3	CUP4	CUP5	CUP6	CUP8
**Ingredients**																
Corn	543	542	541	539	538	536	533	530	579	578	576	574	573	571	569	565
*Candida utilis* protein	0	10.0	20.0	30.0	40.0	50.0	60.0	80.0	0	10.0	20.0	30.0	40.0	50.0	60.0	80.0
Soybean meal	326	317	308	300	291	283	274	258	284	276	267	259	250	242	233	216
Corn protein powder	50.0	50.0	50.0	50.0	50.0	50.0	50.0	50.0	50.0	50.0	50.0	50.0	50.0	50.0	50.0	50.0
Soybean oil	34.0	33.8	33.6	33.4	33.4	33.4	33.4	33.4	43.6	43.6	43.6	43.6	43.6	43.6	43.6	43.6
Dicalcium phosphate	16.0	16.0	15.8	15.6	15.5	15.4	15.2	15.0	14.0	14.0	13.8	13.7	13.6	13.4	13.2	13.0
DL-methionine	1.70	1.70	1.60	1.60	1.60	1.50	1.50	1.40	1.30	1.20	1.20	1.20	1.10	1.10	1.10	1.00
L-lysine	2.50	2.40	2.30	2.20	2.00	1.90	1.80	1.60	1.10	1.00	0.80	0.70	0.60	0.50	0.40	0.20
Limestone	11.3	11.3	11.5	11.6	11.8	11.9	12.1	12.3	10.5	10.5	10.7	10.8	10.9	11.1	11.3	11.5
Titanium dioxide	3.00	3.00	3.00	3.00	3.00	3.00	3.00	3.00	3.00	3.00	3.00	3.00	3.00	3.00	3.00	3.00
Solka flock	0	0	0	1.00	1.00	1.30	2.40	3.20	0	0	0.500	1.00	1.40	1.80	2.30	3.00
Premix^b^	13.0	13.0	13.0	13.0	13.0	13.0	13.0	13.0	13.0	13.0	13.0	13.0	13.0	13.0	13.0	13.0
Total	1,000	1,000	1,000	1,000	1,000	1,000	1,000	1,000	1,000	1,000	1,000	1,000	1,000	1,000	1,000	1,000
**Calculated composition**																
Metabolizable energy, MJ/kg^c^	12.6	12.6	12.6	12.6	12.6	12.6	12.6	12.6	13.0	13.0	13.0	13.0	13.0	13.0	13.0	13.0
Available phosphorus	4.50	4.50	4.50	4.50	4.50	4.50	4.50	4.50	4.00	4.00	4.00	4.00	4.00	4.00	4.00	4.00
**Analyzed composition**																
Dry matter	910	910	910	910	910	910	910	910	910	910	910	910	910	910	920	920
Crude protein	215	213	213	213	214	215	213	215	196	200	200	199	199	199	199	199
Ether extract	69.7	67.3	69.3	68.8	64.7	63.0	68.9	70.4	74.8	76.8	75.4	74.0	76.1	76.7	78.0	78.5
Total phosphorus	7.30	7.30	7.30	7.40	7.40	7.40	7.40	7.40	6.70	6.80	6.70	6.70	6.70	6.80	6.80	6.80
Calcium	10.0	10.0	10.1	9.90	9.90	9.90	9.90	9.90	9.00	9.00	9.00	8.90	8.90	8.90	8.90	8.80
Arginine	13.0	12.6	12.7	12.9	13.0	12.5	12.2	11.9	10.1	10.8	10.4	10.6	10.3	9.90	10.0	9.70
Isoleucine	8.00	7.50	8.00	8.00	8.00	7.90	8.00	7.90	7.00	7.90	7.70	8.00	7.50	7.50	7.60	7.30
Leucine	19.5	19.1	19.1	19.1	19.3	19.3	18.9	18.8	18.5	19.9	19.5	20.2	19.4	19.2	19.5	18.7
Lysine	11.9	11.3	11.8	11.7	11.4	11.4	11.2	11.0	10.1	10.9	10.6	10.7	10.3	10.3	10.3	9.80
Methionine	6.20	6.00	6.30	6.10	6.10	5.30	5.70	5.90	6.60	6.70	6.90	6.40	6.30	6.50	6.20	6.50
Threonine	9.20	9.00	9.00	9.10	9.20	9.30	8.70	8.70	7.40	8.10	8.00	8.40	8.10	8.20	8.40	8.10
Valine	8.80	8.10	8.80	8.70	8.70	8.60	8.40	8.40	7.40	8.10	8.00	8.30	7.90	8.00	8.10	7.80

a CON was the control diet. The other 7 diets were supplemented with *C. utilis* protein at levels of 1%, 2%, 3%, 4%, 5%, 6% and 8%, and were named CUP1, CUP2, CUP3, CUP4, CUP5, CUP6, and CUP8, respectively.

b Supplied per kilogram of diet: Sodium chloride 3g/kg, vitamin A, 8,000 IU, vitamin D_3_ 1,000IU, vitamin E 20 IU, vitamin K_3_ 0.8 mg, vitamin B_1_ 3 mg, vitamin B_2_ 8 mg, vitamin B_6_ 5 mg, vitamin B_12_ 0.02 mg, biotin 0.2 mg, folic acid0.6 mg, pantothenic acid 10 mg, nicotinic acid 40 mg, Cu (as copper sul-fate) 8 mg, Fe (as ferrous sulfate) 100 mg, Mn (as manganese sulfate)120 mg, Zn (as zinc sulfate) 100 mg, I (as potassium iodide) 0.70 mg, Se(as sodium selenite) 0.30 mg, Choline chloride 1500 mg.

c The metabolizable energy was calculated based on the nutritional values of the feedstuffs from the China Feed Database (2020).

### Growth performance and economic evaluation

Body weight and feed consumption were recorded per replicate pen on days 1, 21 and 42, after 8-hour fast. Average daily gain (ADG), average daily feed intake (ADFI), and feed conversion ratio (FCR) were calculated for starter, grower, and overall periods. Broilers were checked every day, and any birds that died or removed was weighed and used to correct for the ADFI and FCR accordingly.

For the economic benefit analysis for broiler feed and products were conducted based on prevailing local market prices and final body weight of broilers at the end of the experimental period. The calculation formulas for each economic indicator are specified as follows: Feed cost per kg of body weight gain (USD/kg gain) = (Diet cost per tonne / 1000) × FCR; Market revenue per bird (USD/bird) = Final live body weight × Market price of live broilers (USD/kg); Net profit per live bird (USD/bird) = Market revenue per bird - Total cost per bird (feed cost + day-old chick cost); Return on investment (ROI) ratio = Net profit per bird in the treatment group / Net profit per bird in the control group.

### Sample collection

To determine CP and amino acid apparent digestibility, broilers had ad libitum access to feed throughout excreta samples collection. The indicator TiO_2_ was used in diets during all period, excreta samples for each cages were collected 2 days before the 21st and 42nd days. Excreta samples were lightly sprayed with 10 % HCl every 12 hours to fix volatile nitrogen and minimize nitrogen loss. Foreign material (feathers, scurf, spilled feed) was carefully removed. After collection, all excreta samples from each pen were mixed well, placed in Petri dishes and dried in a forced-air oven at 65°C. Dried samples were equilibrated for 24 at ambient temperature before weighing, then ground in a mill, passed through a 40-mesh screen (0.45-mm aperture), and stored in airtight bags at -20°C until analysis (Ma et al., 2024). On day 42, one bird per pen closest to the mean body weight (64 birds total) was selected, weighed, and blood samples was collected via jugular venipuncture using anticoagulant-free and heparin sodium vacuum tube. The samples in anticoagulant-free vacuum tube were centrifuged at 3,000 × g for 15 min to obtain serum (Shan et al., 2024). Serum and whole-blood samples were stored at 4°C for later analyses of immunoglobulins, antioxidant indices, pro-inflammatory cytokines, and T-cell subpopulations.

The birds were sacrificed by jugular exsanguination following euthanasia by CO_2_ inhalation. Organs (liver, spleen, bursa of Fabricius, and intestine) were excised intact and weighed to calculate immune organ indices (organ weight relative to live body weight at slaughter, Wang et al., 2025) and abdominal fat rate were determined following the methods specified in NY/T 823-2020 (2020). Cecal digesta samples were collected and snap-frozen in liquid nitrogen for microbiota and mycobiota analysis.

### Apparent digestibility for nutrient

Apparent digestibility of DM, CP, and amino acids was calculated as: Apparent Digestibility (%) = [1 - (TiO₂ in feed / TiO₂ in excreta) × (Nutrient in excreta / Nutrient in feed)] × 100, where TiO₂ in feed and excreta represent absorbance values at 410 nm of feed and excreta samples as dry-matter basis, and nutrient in feed and excreta denote concentrations of the relevant nutrient in feed and excreta as dry-matter basis. Given the lack of experimentally determined metabolizable energy (ME) data for CUP, the ME value of *Saccharomyces cerevisiae*, a species belonging to the same family Saccharomycetaceae as CUP, was adopted as a surrogate value for CUP in the present study.

### Hematological parameters

Collected serum samples were used for the following measurements. Serum immunoglobulins, including immunoglobulin Y (IgY), immunoglobulin A (IgA) and immunoglobulin M (IgM), were determined using a Mindray BS-420 automated biochemistry analyzer (Shenzhen Mindray Bio-Medical Electronics Co., Ltd., Shenzhen, China) with HY-NE456 and HY-753 assay kits (Zhongsheng Beikong Biotechnology Co., Ltd.). Inflammatory cytokines, namely interleukin-2 (IL-2), interleukin-4 (IL-4), interleukin-10 (IL-10), interferon-γ (IFN-γ), C-reactive protein (CRP) and tumor necrosis factor-α (TNF-α), were quantified using commercial ELISA kits (Huaying Biological Technology Research Institute, Beijing, China). For antioxidant parameters, malondialdehyde (MDA) content and the activities of glutathione peroxidase (GSH-Px), superoxide dismutase (SOD), as well as total antioxidant capacity (T-AOC), were assessed using colorimetric assay kits (Nanjing Jiancheng Bioengineering Institute, Nanjing, China). Absorbance values were read at 532 nm for MDA, 412 nm for GSH-Px, 550 nm for SOD, and 405 nm for T-AOC on a Multiskan SkyHigh microplate reader (Thermo Fisher Scientific, China). Collected plasma samples were used for immunophenotyping analysis. Lymphocytes from whole blood were labeled with fluorescent-conjugated monoclonal antibodies targeting chicken CD3⁺ (Mouse Anti-Chicken CD3-AF647, 8200-31), CD4⁺ (Mouse Anti-Chicken CD4-PE, 8210-09), and CD8⁺ (Mouse Anti-Chicken CD8α-AF488, 8220-30) surface markers, and the proportions of T lymphocyte subpopulations were quantified by multicolor flow cytometry using a CytoFLEX S flow cytometer (Beckman Coulter, USA).

### Immunological organ indexes and abdominal fat

The liver, spleen, thymus, intestinal, bursa and abdominal fat, and the organ index was determined as follows:

Organ index (g/kg) = Organ weight (g)/BW (kg).
Abdominal fat percentage (%) = Abdominal fat (g)/ BW (g)

### Composition and diversity of cecum microbiota and mycobiota

Cecal digesta samples were collected from 42-day-old broilers immediately after slaughter, transferred to cryopreservation tubes, and stored at -80°C until intestinal metagenomic analysis. Total genomic DNA was extracted from cecal contents using the TIANMicrobe Magnetic Envir-DNA Kit (Tiangen Biotech, Beijing, China). DNA concentration and purity were determined using a NanoDrop 2000 spectrophotometer (Thermo Fisher Scientific, Waltham, MA, USA), and DNA integrity was verified by 1% agarose gel electrophoresis. High-throughput sequencing was performed by Shanghai Majorbio Bio-Pharm Technology Co., Ltd. (Shanghai, China) on the IlluminaNovaSeq 6000 platform (Illumina, San Diego, CA, USA). Prior to library construction, genomic DNA was sheared to an average insert size of 350 bp using a Covaris M220 focused ultrasonicator (Covaris, Woburn, MA, USA). Paired-end libraries were prepared using the NEXTFLEX Rapid DNA-Seq Kit (Bioo Scientific, Austin, TX, USA) following the manufacturer's instructions. Raw sequencing reads were first processed with fastp (v0.23.0) to remove adapter sequences, low-quality bases (Phred quality score < 20), and reads containing ambiguous bases. To eliminate host DNA contamination, filtered reads were aligned against the birds reference genome (*Gallus gallus* GRCg6a, GCA_000002315.5) using Bowtie2 (v2.4.5), and non-host reads were retained for downstream analysis. De novo assembly of clean reads was performed using MEGAHIT (v1.2.9; Li et al., 2015). Open reading frames (ORFs) were predicted from assembled contigs using Prodigal (v2.6.3; Hyatt et al., 2010) in metagenomic mode. A non-redundant gene catalog was constructed from predicted ORFs using CD-HIT (v4.6.1; Fu et al., 2012) with a 95% sequence identity threshold. Gene abundance profiles were generated by mapping high-quality reads back to the non-redundant catalog using SOAPaligner (v2.21; Li et al., 2008) with a 95% identity cutoff. Taxonomic annotation of non-redundant genes was performed by aligning amino acid sequences against the NCBI non-redundant (NR) protein database using DIAMOND (v0.8.35; Buchfink et al., 2015) with an E-value cutoff of ≤ 1e-5. Functional annotations were obtained by aligning sequences against the Gene Ontology (GO), Kyoto Encyclopedia of Genes and Genomes (KEGG), Carbohydrate-Active enZYmes (CAZy), Comprehensive Antibiotic Resistance Database (CARD), and Pathogen-Host Interactions (PHI) databases using the same parameters. Differential abundance analysis at the taxonomic, functional, and gene levels was conducted using the Kruskal-Wallis test.

### Statistical analysis

Data were collated in Microsoft Excel and analyzed using JMP-pro software (version19.0.0, SAS, Cary, NC). Outliers were removed using the mean ± 3 standard deviation (SD) criterion prior to statistical analysis. All data were subjected to one-way ANOVA, and Tukey's Honest Significant Difference test was used for post hoc comparisons when significant differences (*P* < 0.05) were detected. Linear and quadratic regression analyses were performed to determine the dose-response relationship between dietary CUP levels and growth performance, which served as the key evaluation indicator for optimizing CUP supplementation in broiler diets. Graphical representations of data were generated using GraphPad Prism (v9.5.1; GraphPad Software, San Diego, CA, USA). For metagenomic analysis, the control group, the group with the best growth performance, and the group with the worst growth performance were selected as typical representatives to analyze the differences in cecal microbiota among the three groups. Principal coordinate analysis (PCoA) was performed and visualized using R (v3.2.5). Differentially abundant features were identified using linear discriminant analysis effect size (LEfSe) with a significance threshold of *P* < 0.05 and a linear discriminant analysis (LDA) score > 3.0.

Specifically, a quadratic regression model was constructed using the quadratic regression procedure to further elucidate the dose-response relationship between dietary CUP levels and growth performance, the primary endpoint for determining optimal CUP inclusion rates. The quadratic regression model was defined as:

$$Y=aX^{2}+bX+c$$

where Y is the measured value of the growth performance indicator, *X* is the dietary CUP supplementation level (%), a is the quadratic coefficient, b is the linear coefficient, and c is the constant term. The optimal CUP supplementation level was calculated as the axis of symmetry of the quadratic curve: *X*_0_=b/−2a.

## Results

### Comparison of nutritional composition between *Candida utilis* protein and soybean meal

Table 1 compares the nutritional composition of CUP and SBM. The crude protein content of CUP was 38.30%, and the crude fat content was 6.67%, which was markedly higher than that of SBM (1.50%). The total phosphorus content of CUP (1.62%) was higher than that of SBM (0.78%), and the gross energy of CUP (4,532 kcal/kg) was also higher than that of SBM (4,190 kcal/kg). In addition, the anti-nutritional factors in the yeast cell wall of CUP included β-glucan at 23.60% and mannan at 5.10%.

### Growth performance and economic analysis

The effects of graded dietary CUP supplementation on broiler growth performance are presented in Table 3. During the starter phase (d 1-21), FBW, ADG, ADFI, and FCR all exhibited significant quadratic responses with increasing dietary CUP inclusion (*P* < 0.001). CON group, broilers fed diets containing 1% to 5% CUP (CUP1 to CUP5) showed no significant differences in these growth parameters. In contrast, supplementation with 6% and 8% CUP (CUP6 and CUP8) significantly reduced FBW, ADG, and ADFI and increased FCR (*P* < 0.001) relative to the CON group. During the grower phase (d 22-42), FBW, ADG, and ADFI showed significant quadratic responses to rising dietary CUP levels. Relative to the CON group, 42-day FBW was significantly decreased in both the CUP6 and CUP8 groups (*P* < 0.001). ADG was significantly lower in the CUP6 group (*P* = 0.001), and ADFI was significantly reduced in the CUP8 group (*P* < 0.001). No significant difference in FCR was observed across all experimental groups (*P* > 0.05). Over the entire experimental period (d 1-42), ADG and ADFI displayed highly significant quadratic responses to increasing dietary CUP levels (*P* < 0.001). Compared with the CON group, both ADG and ADFI were significantly decreased in the CUP8 group (*P* < 0.001), and ADG was also significantly reduced in the CUP6 group (*P* < 0.001). Although no significant difference in overall FCR was detected among all treatments (*P* > 0.05), FCR exhibited a significant quadratic response to dietary CUP supplementation (*P* = 0.004).

**Table 3:** Effects of SBM replacement by CUP on growth performance of broilers^a^.

Items^b^	Groups								SEM^c^	*P*-value	Polynomialcontrasts	
	CON	CUP1	CUP2	CUP3	CUP4	CUP5	CUP6	CUP8			Linear	Quadratic
Days 1-21												
IBW at 1d, g	45.0	45.4	45.0	45.1	45.0	45.1	44.6	44.7	0.130	0.889	0.208	0.403
FBW at 21d, g	766^d^^d^	822^d^	822^d^	800^d^^d^	827^d^	704^d^^d^	635^d^	612^d^	12.4	<0.001	<0.001	<0.001
1-21d ADG, g/d	34.3^d^^d^	37.0^d^	37.0^d^	36.0^d^^d^	37.2^d^	31.4^d^^d^	28.1^d^	27.0^d^	0.590	<0.001	<0.001	<0.001
1-21d ADFI, g/d	45.4^d^^d^	47.9^d^	48.0^d^	46.2^d^^d^	48.5^d^	41.3^d^^d^	39.7^d^	38.1^d^	0.630	<0.001	<0.001	<0.001
1-21d FCR	1.32^d^	1.30^d^	1.30^d^	1.29^d^	1.30^d^	1.31^d^	1.42^d^	1.41^d^	0.008	<0.001	<0.001	<0.001
Days 22-42												
FBW at 42d, g	2538^d^^d^	2632^d^^d^	2647^d^^d^	2603^d^^d^	2673^d^	2428^d^^d^	2266^d^	2205^d^	27.2	<0.001	<0.001	<0.001
22-42d ADG, g/d	83.8^d^^d^^d^	86.2^d^^d^	86.9^d^	85.8^d^^d^^d^	87.9^d^	82.1^d^^d^^d^	77.7^d^	78.4^d^^d^	0.750	0.001	0.003	<0.001
22-42d ADFI, g/d	139^d^^d^	143^d^	142^d^	140^d^^d^	145^d^	136^d^^d^^d^	128^d^^d^	123^d^	1.27	<0.001	<0.001	<0.001
22-42d FCR	1.66	1.66	1.67	1.65	1.65	1.65	1.66	1.64	0.009	0.995	0.548	0.093
Days 1-42												
1-42d ADG, g/d	60.3^d^^d^	62.5^d^^d^	62.9^d^^d^	61.8^d^^d^	63.5^d^	57.7^d^^d^	53.8^d^	52.4^d^	0.650	<0.001	<0.001	<0.001
1-42d ADFI, g/d	91.9^d^^d^^d^	95.3^d^^d^	94.2^d^^d^	93.0^d^^d^	96.8^d^	88.3^d^^d^^d^	83.8^d^^d^	80.6^d^	0.900	<0.001	<0.001	<0.001
1-42d FCR	1.53	1.52	1.53	1.51	1.52	1.53	1.57	1.55	0.006	0.724	0.207	0.004

a CON was the control diet. The other 7 diets were supplemented with *C. utilis* protein at levels of 1%, 2%, 3%, 4%, 5%, 6% and 8%, and were named CUP1, CUP2, CUP3, CUP4, CUP5, CUP6, and CUP8, respectively.

b IBW, Initial body weight; FBW, final body weight; ADG, average daily gain; ADFI, average daily feed intake; FCR, feed conversion ratio.

c SEM, standard error of the mean.

d Means with different superscripts in the same row are significantly different.

As shown in Table 4, quadratic regression analysis using body weight and FCR as evaluation indicators revealed that the optimal dietary inclusion range of CUP for broilers was 1.6% to 2.5%.

**Table 4:** Regression analysis between *CUP* addition levels in diet and broiler growth performance.

Item	Quadratic Regression equations	*R*^b^	*P*-value	*Candida utili* protein optimal level / %
1-21d BW^a^	*Y*=7.94E2+18.29*X*-5.7*X*^b^	0.511	<0.001	1.60
1-21d FCR^b^	*Y*=1.31-0.02*X*+4.01E-3*X*^b^	0.448	<0.001	2.50
22-42d BW	*Y*=1.84E3+24.88*X*-6.74*X*^b^	0.320	<0.001	1.85
22-42d FCR	-	0.007	0.882	-
1-42d BW	*Y*=2.59E3+43.17*X*-12.43*X*^b^	0.461	<0.001	1.74
1-42d FCR	*Y*=1.53-3.92E-3*X*+9.48E-4*X*^b^	0.042	0.294	2.10

a BW, body weight.

b FCR, (feed conversion ratio) = feed intake/body weight gain.

Economic analysis was performed according to the established calculation formulas. Feed cost per kilogram of body weight gain was 1.35 USD/kg for the CON group and 0.01 USD/kg for the CUP4 group. Market revenue per bird reached 2.44 USD in the CON group and 2.57 USD in the CUP4 group. Net profit per live bird was 0.30 USD for the CON group, compared with 0.35 USD for the CUP4 group. The ROI of the CUP4 group relative to the CON control was 1.17.

### Apparent digestibility of nutrient

The apparent digestibility of nutrient in broilers fed graded levels of CUP is presented in the Table 5, Table 6. Significant differences were observed among treatments for the digestibility of CP, DM, and most amino acids across both feeding phases. During the starter period (d 1-21), the digestibility of DM and CP exhibited a quadratic response (*P* < 0.001) to increasing CUP inclusion, rising to a peak at CUP4 before declining. The CUP4 showed significantly higher DM and CP digestibility than the CUP8 (*P* < 0.001). Among essential amino acids (EAA), digestibility was generally highest in the CUP4. Total amino acid (TAA) digestibility was significantly greater in the CUP4 compared with the control and CUP8 (*P* < 0.001). In the Table 5, notably, methionine digestibility declined linearly with CUP addition, decreasing from 93.70 % in the control to 79.00 % in the CUP8 (*P* < 0.001). In the grower period (Table 6), DM digestibility was significantly influenced by treatment (*P* = 0.003), with the CUP3 recording the highest value. CP digestibility was significantly higher in the CUP4 than in the control (*P* < 0.05), whereas the CUP8 showed the lowest CP digestibility. For most EAA and non-essential amino acids (NEAA), digestibility was improved with moderate yeast inclusion (3-6%), with several reaching their peaks in these groups. Consequently, the TAA digestibility was significantly higher in all yeast-supplemented groups than in the control group (*P* < 0.001).

**Table 5:** Effects of SBM replacement by CUP on **apparent** digestibility of broilers during the starter phase (d 19-20)^a^.

Apparent digestibility %	Starter diets								SEM^b^	*P*-value	Polynomial contrasts	
	CON	CUP1	CUP2	CUP3	CUP4	CUP5	CUP6	CUP8			Linear	Quadratic
Dry matter	78.0^c^	78.9^c^^c^	79.1^c^^c^	79.3^c^	79.8^c^	78.0^c^	76.2^c^	76.3^c^	0.171	<0.001	<0.001	<0.001
Crude protein	71.6^c^	72.3^c^	72.0^c^	72.3^c^	72.5^c^	71.5^c^	68.9^c^	68.2^c^	0.244	<0.001	<0.001	<0.001
Total amino acids	88.0^c^^c^	88.2^c^^c^	88.9^c^^c^^c^	89.2^c^^c^	89.6^c^	88.7^c^^c^^c^	88.1^c^^c^	87.0^c^	0.125	<0.001	0.414	0.414
**Essential amino acids**												
Arginine	91.4^c^	92.6^c^^c^	92.4^c^^c^^c^	92.3^c^^c^^c^	92.9^c^	92.7^c^^c^	92.3^c^^c^^c^	91.7^c^^c^	0.100	0.001	0.334	0.334
Histidine	87.9^c^	88.3^c^^c^	89.4^c^^c^	88.9^c^^c^	91.4^c^	90.7^c^	90.8^c^	90.5^c^^c^	0.170	<0.001	<0.001	<0.001
Isoleucine	85.1^c^^c^	83.7^c^^c^	85.8^c^^c^	86.7^c^	86.2^c^	84.9^c^^c^^c^	84.8^c^^c^^c^	82.6^c^	0.240	<0.001	0.237	0.328
Leucine	90.6^c^	90.6^c^	91.3^c^^c^	92.3^c^	92.5^c^	92.2^c^	91.8^c^^c^	90.7^c^	0.140	<0.001	0.013	0.013
Lysine	91.6^c^^c^	92.0^c^	92.0^c^	91.9^c^	92.4^c^	91.6^c^^c^	90.9^c^^c^	90.5^c^	0.100	<0.001	<0.001	<0.001
Methionine	93.7^c^	93.5^c^	94.1^c^	93.5^c^	91.3^c^	90.4^c^	88.2^c^	79.0^c^	0.540	<0.001	<0.001	<0.001
Phenylalanine	90.3^c^^c^	90.1^c^	91.1^c^^c^^c^	91.8^c^	92.0^c^	91.5^c^	91.4^c^^c^	90.9^c^^c^^c^	0.120	<0.001	<0.001	<0.001
Threonine	79.3^c^^c^	80.8^c^	80.9^c^	80.6^c^^c^	81.1^c^	78.8^c^	76.6^c^	75.6^c^	0.260	<0.001	<0.001	<0.001
Valine	86.7^c^^c^	86.6^c^^c^	87.8^c^^c^	88.1^c^	88.0^c^^c^	86.6^c^^c^	86.2^c^	84.7^c^	0.160	<0.001	0.010	0.003
**Nonessential amino acids**												
Alanine	82.3^c^^c^	87.2^c^	88.2^c^^c^^c^	89.1^c^	89.4^c^	88.9^c^^c^	88.8^c^^c^	87.2^c^	0.160	<0.001	0.027	0.087
Asparagine	87.6^c^	87.8^c^	88.3^c^^c^	88.7^c^^c^	89.5^c^	88.6^c^^c^	88.3^c^^c^	87.6^c^	0.120	<0.001	0.105	0.105
Glycine	80.4^c^	81.7^c^^c^	82.9^c^^c^	82.8^c^^c^	84.6^c^	83.1^c^	82.6^c^^c^	81.1^c^	0.180	<0.001	0.012	0.040
Glutamate	91.1^c^	91.2^c^^c^	92.1^c^^c^^c^^c^	92.6^c^^c^	93.0^c^	92.5^c^^c^	92.3^c^^c^^c^	91.7^c^^c^^c^	0.110	<0.001	0.001	0.001
Cysteine	81.4^c^^c^	81.8^c^^c^	81.8^c^^c^	82.4^c^	82.3^c^	81.0^c^^c^	81.8^c^^c^	79.6^c^	0.200	0.038	0.093	0.171
Tyrosine	86.3^c^^c^^c^	86.7^c^^c^^c^	88.2^c^	87.8^c^^c^	86.8^c^^c^^c^	85.4^c^^c^	85.8^c^^c^	84.8^c^	0.180	<0.001	<0.001	0.002
Proline	81.1^c^^c^	80.8^c^^c^	81.3^c^^c^	81.5^c^	81.6^c^	79.7^c^	77.6^c^^c^	76.1^c^^c^	0.250	<0.001	<0.001	<0.001
Serine	86.8^c^^c^	87.6^c^^c^^c^	88.1^c^^c^^c^	88.6^c^^c^	89.2^c^	88.1^c^^c^^c^	87.5^c^^c^^c^	86.4^c^	0.140	<0.001	0.834	0.829

a CON was the control diet. The other 7 diets were supplemented with *C. utilis* protein at levels of 1%, 2%, 3%, 4%, 5%, 6% and 8%, and were named CUP1, CUP2, CUP3, CUP4, CUP5, CUP6, and CUP8, respectively.

b SEM, standard error of the mean.

c Means with different superscripts in the same row are significantly different.

**Table 6:** Effects of SBM replacement by CUP on **apparent** digestibility of broilers during the grower phase (d 40-41)^a^.

Apparent digestibility %	Grower diets									SEM^b^	*P*-value	Polynomial contrasts	
	CON		CUP1	CUP2	CUP3	CUP4	CUP5	CUP6	CUP8			Linear	Quadratic
Dry matter	80.8^c^	81.4^c^^c^		81.5^c^^c^	83.6^c^	82.6^c^^c^	82.1^c^^c^	82.7^c^^c^	82.7^c^^c^	0.201	0.003	0.004	0.010
Crude protein	68.3^c^^c^	65.9^c^^c^		65.2^c^^c^	67.2^c^^c^	69.6^c^	67.4^c^^c^	63.3^c^^c^	62.4^c^	0.588	0.043	0.088	0.235
Total amino acids	89.8^c^	91.4^c^		91.5^c^	92.7^c^	92.1^c^	92.3^c^	92.3^c^	91.8^c^	0.164	<0.001	<0.001	<0.001
**Essential amino acids**													
Arginine	94.9^c^	96.0^c^^c^		95.8^c^^c^	96.3^c^	95.9^c^^c^	96.0^c^^c^	96.5^c^	96.5^c^	0.110	0.005	<0.001	<0.001
Histidine	93.6^c^	94.6^c^^c^		94.6^c^^c^	95.6^c^	95.0^c^	95.2^c^	95.8^c^	95.9^c^	0.130	<0.001	<0.001	<0.001
Isoleucine	85.1^c^^c^	83.7^c^^c^		85.8^c^^c^	86.7^c^	86.2^c^	84.9^c^^c^^c^	84.8^c^^c^^c^	82.6^c^	0.330	<0.001	0.001	0.003
Leucine	92.9^c^	93.6^c^		93.7^c^^c^	95.2^c^	94.5^c^	94.3^c^	94.2^c^^c^	93.9^c^	0.160	0.011	0.030	0.030
Lysine	91.6^c^	92.0^c^		92.0^c^^c^	91.9^c^	92.4^c^	91.6^c^	90.9^c^	90.5^c^	0.150	<0.001	<0.001	<0.001
Methionine	81.2	81.4		82.2	81.9	81.8	82.5	80.8	82.3	0.200	0.535	0.378	0.354
Phenylalanine	92.8^c^	94.1^c^^c^		94.0^c^^c^	95.1^c^	94.3^c^	94.3^c^	94.6^c^	94.4^c^	0.130	<0.001	0.002	0.002
Threonine	85.4^c^	88.6^c^^c^		89.1^c^	90.3^c^	89.5^c^	90.3^c^	90.9^c^	90.5^c^	0.320	<0.001	<0.001	<0.001
Valine	86.9^c^	88.2^c^^c^		88.2^c^^c^	90.8^c^	89.6^c^^c^	89.7^c^^c^	88.6^c^^c^	87.5^c^	0.290	0.007	0.175	0.142
**Nonessential amino acids**													
Alanine	87.6^c^^c^	88.9^c^^c^^c^		87.9^c^^c^^c^	91.0^c^	89.4^c^^c^	89.6^c^^c^	88.6^c^^c^^c^	85.9^c^	0.290	<0.001	0.747	0.800
Asparagine	89.1^c^	90.5^c^^c^		91.3^c^	92.2^c^	91.5^c^	92.1^c^	91.8^c^	90.9^c^	0.180	<0.001	<0.001	<0.001
Glycine	85.1^c^	87.4^c^^c^		87.5^c^^c^	89.4^c^	88.4^c^	88.9^c^	88.8^c^	87.5^c^^c^	0.270	0.001	0.003	0.007
Glutamate	91.6^c^	93.6^c^		93.7^c^	94.4^c^	93.9^c^	94.5^c^	94.7^c^	94.5^c^	0.160	<0.001	<0.001	<0.001
Cysteine	88.8^c^	92.2^c^		91.3^c^^c^	94.4^c^	92.6^c^	93.3^c^	93.5^c^	92.9^c^	0.310	<0.001	<0.001	<0.001
Tyrosine	87.0^c^	90.2^c^^c^		89.5^c^^c^	90.7^c^^c^	90.2^c^^c^	90.0^c^^c^	92.0^c^	91.7^c^	0.330	0.006	<0.001	<0.001
Proline	87.4^c^	88.7^c^^c^		89.0^c^^c^	89.3^c^^c^	90.3^c^	89.8^c^	89.7^c^	89.1^c^^c^	0.200	0.005	0.001	0.005
Serine	90.6^c^	92.7^c^^c^		92.4^c^^c^	93.4^c^	92.7^c^^c^	93.3^c^	93.7^c^	93.6^c^	0.200	<0.001	<0.001	<0.001

a CON was the control diet. The other 7 diets were supplemented with *C. utilis* protein at levels of 1%, 2%, 3%, 4%, 5%, 6% and 8%, and were named CUP1, CUP2, CUP3, CUP4, CUP5, CUP6, and CUP8, respectively.

b SEM, standard error of the mean.

c Means with different superscripts in the same row are significantly different.

### Relative organ weights, immune indices, and antioxidant capacity

Table 7 presents the relative weights of immune organs and abdominal fat content in broilers. Dietary CUP supplementation did not significantly affect the relative weights of the liver, thymus, intestine, spleen, or bursa of Fabricius, and had no significant impact on abdominal fat content (*P* > 0.05).The effects of dietary CUP on serum and plasma immune parameters, as well as antioxidant capacity, are summarized in Table 8, Table 9.

**Table 7:** Effects of SBM replacement by CUP on relative weights of immune organs and abdominal fat percentage of broilers^a^.

Item, g/kg	Groups								SEM^b^	*P*-value	Polynomial contrasts	
	CON	CUP1	CUP2	CUP3	CUP4	CUP5	CUP6	CUP8			Linear	Quadratic
Liver	26.7	25.6	27.4	24.2	24.0	24.0	24.4	25.7	0.440	0.312	0.105	0.254
Thymus	2.50	2.20	2.40	2.40	2.30	2.60	2.80	2.50	0.070	0.608	0.334	0.626
Intestinal	27.4	24.9	25.4	25.6	23.5	25.0	25.8	27.3	0.390	0.208	0.705	0.713
Spleen	1.50	1.40	1.70	1.60	1.60	1.90	1.70	2.10	0.050	0.261	0.010	0.019
Bursa	0.800	0.700	1.00	1.00	0.800	0.800	0.900	0.900	0.050	0.729	0.950	0.824
Abdominal fat, %	1.20	1.00	1.00	1.00	1.10	1.20	1.40	1.20	0.040	0.112	0.059	0.159

a CON was the control diet. The other 7 diets were supplemented with *C. utilis* protein at levels of 1%, 2%, 3%, 4%, 5%, 6% and 8%, and were named CUP1, CUP2, CUP3, CUP4, CUP5, CUP6, and CUP8, respectively.

b SEM, standard error of the mean.

**Table 8:** Effects of SBM replacement by CUP on Serum immune parameters^a^.

Item^b^	Groups								SEM^c^	*P*-value	Polynomial contrasts	
	CON	CUP1	CUP2	CUP3	CUP4	CUP5	CUP6	CUP8			Linear	Quadratic
IgY, g/L	4.40^d^	4.60^d^	4.70^d^^d^	4.80^d^^d^	4.90^d^^d^	5.00^d^^d^	5.10^d^^d^	5.70^d^	0.090	0.022	<0.001	0.002
IgM, g/L	1.50^d^	1.50^d^	1.60^d^^d^	1.70^d^^d^	1.70^d^^d^	1.70^d^^d^	1.80^d^^d^	1.90^d^	0.020	0.006	<0.001	<0.001
IgA, g/L	2.00^d^	2.10^d^^d^	2.20^d^^d^	2.20^d^^d^	2.30^d^^d^^d^	2.40^d^^d^^d^	2.50^d^^d^	2.60^d^	0.040	0.002	<0.001	<0.001
CD3^+^, %	1.60	1.10	1.30	1.50	2.00	1.70	1.20	1.30	0.080	0.053	0.948	0.621
CD4^+^, %	0.800	0.700	0.800	0.800	1.10	1.00	0.600	0.700	0.050	0.077	0.874	0.504
CD8^+^, %	0.500	0.300	0.300	0.300	0.500	0.400	0.300	0.300	0.200	0.052	0.518	0.801
CD4^+^/CD8^+^, %	2.10	2.50	3.40	2.60	2.50	2.60	2.60	2.60	0.130	0.417	0.288	0.230
CRP, mg/L	26.3	25.6	24.0	24.5	23.7	23.7	22.3	21.1	0.500	0.208	0.002	0.009
**T helper type 1**												
IL-2, pg/mL	232^d^	214^d^	208^d^^d^	193^d^	172.^d^	167^d^	161^d^	156^d^	3.44	<0.001	<0.001	<0.001
TNF-αpg/mL	46.1^d^	42.8^d^^d^	39.6^d^^d^	37.0^d^^d^	33.9^d^^d^	30.7^d^^d^	28.4^d^	25.0^d^	0.850	<0.001	<0.001	<0.001
IFN-γpg/mL	36.4^d^	35.6^d^^d^	33.8^d^^d^^d^	32.9^d^^d^^d^	31.2^d^^d^^d^	29.1^d^^d^	28.2^d^	27.5^d^	0.670	0.001	<0.001	<0.001
**T helper type 2**												
IL-4, pg/mL	5.90^d^	6.20^d^	6.20^d^	7.10^d^^d^	6.90^d^^d^	7.70^d^	7.80^d^	8.00^d^	0.140	<0.001	<0.001	<0.001
IL-10, pg/mL	11.8^d^	11.6^d^	12.1^d^^d^	13.0^d^^d^^d^	12.8^d^^d^	13.8^d^^d^	14.6^d^	14.7^d^	0.210	<0.001	<0.001	<0.001

a CON was the control diet. The other 7 diets were supplemented with *C. utilis* protein at levels of 1%, 2%, 3%, 4%, 5%, 6% and 8%, and were named CUP1, CUP2, CUP3, CUP4, CUP5, CUP6, and CUP8, respectively.

b IgY, Immunoglobulin-Y; IgA, Immunoglobulin-A; IgM, Immunoglobulin-M; IL-2, Interleukin-2; IL-4, Interleukin-4; IL-10, Interleukin-10; IFN-γ, Interferon gamma; CRP, C-reactive protein; TNF-α Tumor necrosis factor α.

c SEM, standard error of the mean.

d Means with different superscripts in the same row are significantly different.

**Table 9:** Effects of SBM replacement by CUP on serum antioxidant capacity in broilers^a^.

Item, U/mL	Groups								SEM^c^	*P*-value	Polynomial contrasts	
	CON	CUP1	CUP2	CUP3	CUP4	CUP5	CUP6	CUP8			Linear	Quadratic
SOD^b^	54.7^d^	61.1^d^	67.3^d^	70.9^d^^d^	76.3^d^^d^	79.3^d^^d^	83.3^d^	89.6^d^	1.38	<0.001	<0.001	<0.001
GSH-Px	124^d^	138^d^	151^d^	162^d^^d^	168^d^^d^	176^d^^d^	183^d^	200^d^	2.85	<0.001	<0.001	<0.001
T-AOC	10.9^d^	12.1^d^^d^	13.1^d^^d^	13.5^d^^d^	14.4^d^^d^^d^	15.0^d^^d^	15.6^d^^d^	16.8^d^	0.240	<0.001	<0.001	<0.001
MDA, noml/mL	5.20^d^	4.60^d^^d^	4.50^d^^d^^d^	4.20^d^^d^^d^	3.80^d^^d^^d^	3.50^d^^d^^d^	3.40^d^^d^	2.90^d^	0.100	<0.001	<0.001	<0.001

a CON was the control diet. The other 7 diets were supplemented with *C. utilis* protein at levels of 1%, 2%, 3%, 4%, 5%, 6% and 8%, and were named CUP1, CUP2, CUP3, CUP4, CUP5, CUP6, and CUP8, respectively.

b SOD, Superoxide dismutase; GSH-Px, Glutathione peroxidase; T-AOC Total antioxidant capacity; MDA, Malondialdehyde.

c SEM, standard error of the mean.

d Means with different superscripts in the same row are significantly different.

As shown in Table 8, serum concentrations of immunoglobulins (IgY, IgA, IgM) and pro-inflammatory cytokines (IL-2, TNF-α, IFN-γ) exhibited significant quadratic responses to increasing dietary CUP levels (*P* < 0.01). Immunoglobulin concentrations rose significantly with higher CUP inclusion (*P* < 0.05), with values in the CUP8 group significantly greater than those in the CON group. By contrast, pro-inflammatory cytokine concentrations decreased markedly with increasing dietary CUP levels (*P* < 0.001). In parallel, the concentrations of anti-inflammatory cytokines IL-4 and IL-10 increased significantly in a dose-dependent manner (*P* < 0.001).

For antioxidant capacity (detailed in Table 9), the activities of SOD and GSH-Px, MDA content, and T-AOC all showed highly significant quadratic responses to graded dietary CUP levels (*P* < 0.001). Compared with the CON group, SOD and GSH-Px activities and T-AOC increased linearly with rising CUP supplementation (*P* < 0.001), while MDA content followed a significant linear decrease (*P* < 0.001).

### Microbiome analysis

Analysis of alpha-diversity indices is presented in Fig. 1A. One replicate from the CUP4 group and two replicates from the CUP8 group were removed as outliers based on the mean ± 3 SD criterion. The Chao1, Shannon, and Simpson indices were calculated, and no significant differences were observed among the CON, CUP4, and CUP8 groups. However, numerical decreases in microbial richness and evenness were noted in the CUP8 group, indicating potential concentration of dominant taxa. β-diversity analysis conducted via principal coordinate analysis (PCoA) and verified by 3D-PCoA (Fig. 1B) revealed clear separation of microbial community structures among the three groups (analysis of similarities, ANOSIM: R = 0.201, *P* = 0.001).

**Fig. 1:**
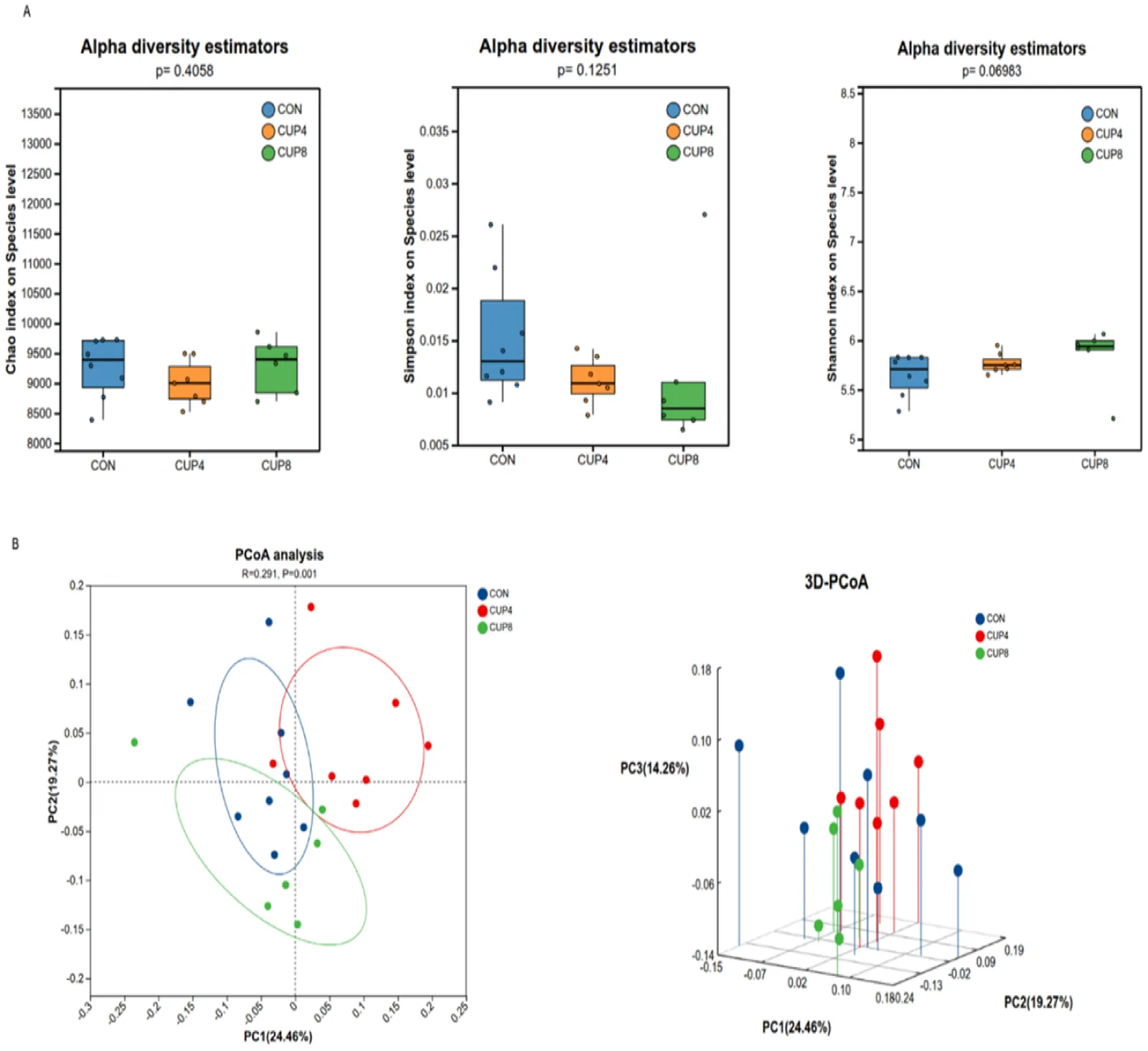
**Effects of SBM replacement by CUP on microbiota diversity in broilers.** (A) Alpha diversity indices on species level, including Chao1, Simpson, and Shannon estimators. (B) Beta diversity analysis visualized by Principal Coordinate Analysis (PCoA) and 3D-PCoA based on the species level (ANOSIM, R = 0.201, P = 0.001). CON was the control diet, n = 8; CUP = *C. utilis* protein. CUP4 was 4% were supplemented with *C. utilis* protein, n = 7; CUP8 was 8% were supplemented with *C. utilis* protein, n = 6.

The effects of dietary CUP on the abundance of intestinal microbiota at the genus level are presented in Fig. 2A. The core microbiota across all treatment groups was dominated by *Alistipes, Bacteroides, Mediterraneibacter*, and *Ligilactobacillus*, with *Mediterraneibacter* showing significant differences among groups (*P* < 0.05). Notably, increasing dietary CUP levels led to an increase in *Bacteroides* abundance, and the CUP4 group had higher *Gemmiger* abundance than the other two groups. The analysis of species-level functional contributions (Fig. 2B) and species abundances (Tables S1 and S2) showed that, compared with the CON group, the CUP8 group exhibited significantly higher relative contributions of *Anaeromassilibacillus* to both metabolic pathways and cellular processes (*P*<0.001). In contrast, the relative contribution of *Blautia* was significantly lower in the CUP8 group for both metabolic pathways (*P*=0.043) and cellular processes (*P*=0.036). The relative contribution of *Clostridium* to cellular processes was significantly higher in the CUP4 group (*P*=0.040). In addition, the relative contribution of *Streptococcus* was significantly lower in both the CUP4 and CUP8 groups for metabolic pathways (*P*=0.007) and cellular processes (*P*=0.006).

**Fig. 2:**
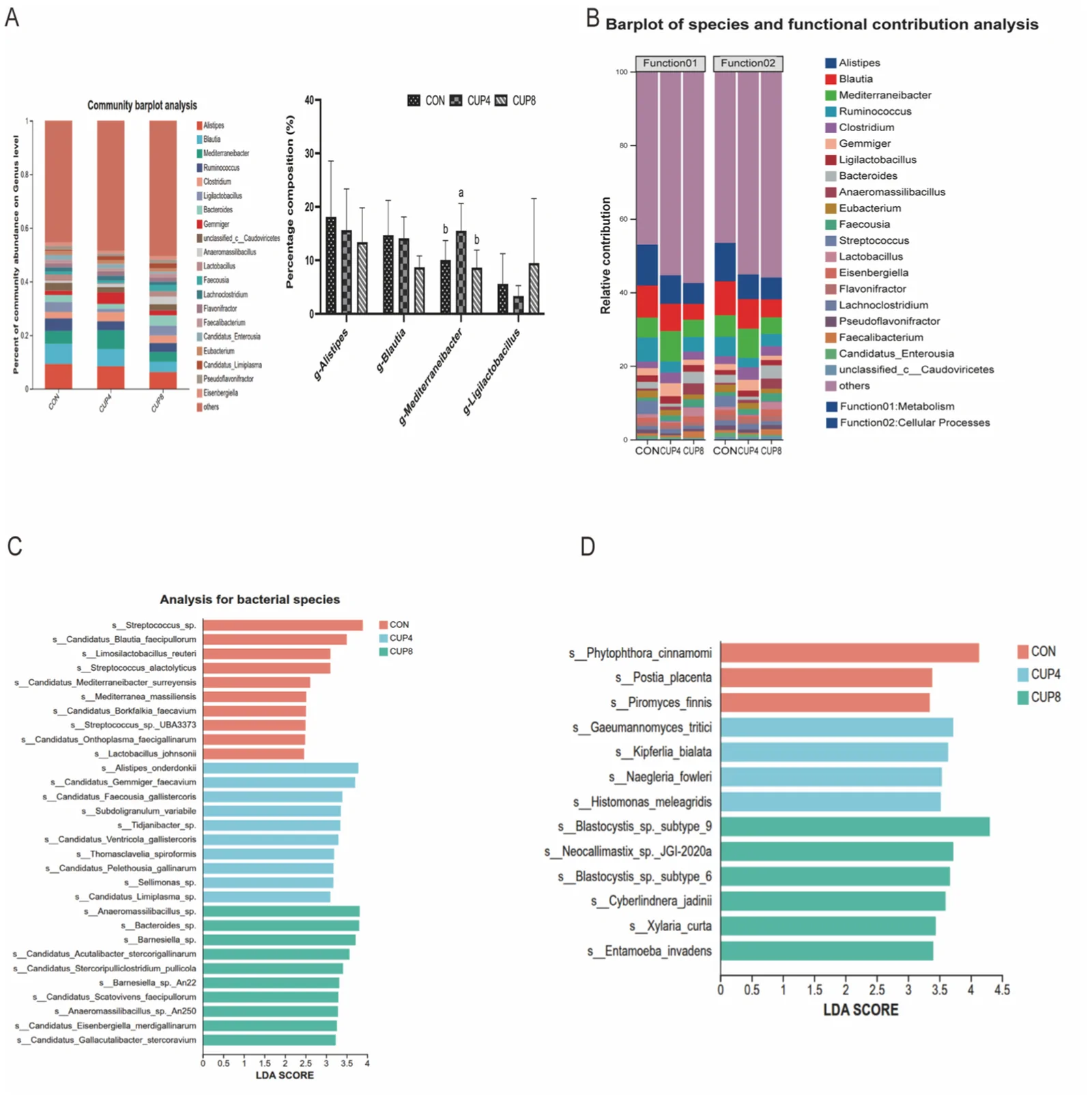
**Effects of SBM replacement by CUP on cecal microbiota and mycobiota in broilers.** (A) Changes in microbiota at the genus level. (B) Bar plot of species and functional contribution analysis. (C) Linear Discriminant Analysis Effect Size (LEfSe) analysis for bacterial species; with an LDA score threshold > 3.0. (D) Linear Discriminant Analysis Effect Size (LEfSe) analysis for eukaryotic species with an LDA score threshold > 3.0. ^a,b^ Bars with different letters are significantly different, Turkey test, *P*< 0.05, mean ± SEM (standard error of treatment means), CON was the control diet, n = 8; CUP = *C. utilis* protein. CUP4 was 4% were supplemented with *C. utilis* protein, n = 7; CUP8 was 8% were supplemented with *C. utilis* protein, n = 6.

LEfSe identified treatment-specific biomarkers at the species level (Fig. 2C). The control group was characterized by high abundances of fermentative commensal bacteria, particularly *Streptococcus* sp, *Limosilactobacillus reuteri*, and *Candidatus Blautia faecipullorum*. In contrast, the CUP4 group was enriched in members of the Bacteroidetes and Firmicutes phyla, such as *Alistipes onderdonkii* and *Candidatus gemmiger faecavium*. The CUP8 group showed a distinct shift toward strict anaerobes, including *Anaeromassilibacillus* sp and *Bacteroides* sp, reflecting changes in dominant taxa at different supplementation levels. Parallel successional changes were also observed in the eukaryotic community (Fig. 2D). Saprophytic fungi were enriched in the CON, while parasitic protists were more prevalent in the CUP4 group. Most notably, the CUP8 group was dominated by the supplemented CUP and anaerobic gut fungi.

## Discussion

Numerous studies have confirmed that yeast-derived microbial proteins improve broiler growth performance and intestinal health, but variations in strains, fermentation processes, and purification methods result in poor comparability of results. As a type of single-cell protein, CUP has advantages such as higher crude fat content and shorter production cycle compared with soybean meal. Its cell wall polysaccharides can regulate animal immunity and intestinal health, making it a high-quality alternative raw material for soybean meal reduction and substitution. The CUP used in this study its optimal supplementation level in broiler diets remains unstandardized. Combining the model-derived range and empirical peak value allows precise determination of the CUP supplementation threshold. These findings align with Cruz et al. (2020), who reported no adverse effects on performance when substituting CUP for 5% soybean meal. While their study established a baseline at 5% replacement, our systematic titration demonstrates that 4% inclusion optimizes growth and nutrient utilization. Conversely, higher inclusion levels depressed body weight, primarily due to thereduction of feed intake. This decline is likely attributable to reduced diet palatability (Grigore et al., 2025) and the anti-nutritional effects of yeast cell-wall non-starch polysaccharides (NSPs), specifically β-glucans and mannans (Kogan & Kocher, 2007). With respect to palatability, the high nucleic acid content of yeast biomass is another key factor influencing palatability. The nucleic acid content of yeast protein is approximately 3-9% (Edozien et al., 1970; Castro et al., 1971; Zee and Simard, 1975) on a dry matter basis. Metabolism of nucleic acids produces uric acid (Cruz et al., 2020), which increases metabolic burden in young chicks and indirectly suppresses feed intake at high inclusion levels. Furthermore, while nucleic acids function as umami-tasting compounds, excessive levels reduce feed preference in broilers (Yoshida et al., 2022). Additionally, the fine particulate nature of yeast biomass in CUP alters feed pellet texture. Relative to conventional protein sources, the yeast fraction generates greater friction and elevated temperatures within the pellet mill during processing, which modifies pellet durability index and in turn impairs feed palatability (Cruz et al., 2020). Although β-glucan concentrations in the CUP8 diet remained below the growth-inhibitory threshold reported for cereal-derived β-glucans (Choct et al., 1990), growth performance was still impaired. This observation indicates that the biological impact of NSPs is shaped by molecular structure as much as by concentration. Unlike the linear β-(1,3)-(1,4) polymers present in cereals, yeast β-glucans are insoluble, branched structures consisting of β-(1,3) and β-(1,6) linkages that function primarily as immunomodulators (Yu et al., 2018). Furthermore, in vitro evidence that exogenous β-(1,3)-glucanase and β-mannanase can effectively degrade CUP cell wall components (Fig. S1) suggests that enzyme supplementation may hydrolyze β-glucan and mannan, thereby mitigating the anti-nutritional properties of CUP.

Beyond feed intake limitations, high CUP levels altered nutrient utilization. While most nutrient digestibilities peaked at 4%, but in starter phase methionine digestibility declined linearly with increasing inclusion. Given that methionine is the first limiting amino acid in poultry, the drop from 93.70% (control) to 79.00% (CUP8) represents a critical nutritional bottleneck, likely caused by yeast cell-wall components hindering amino acid absorption (Rodríguez et al., 2013). Therefore, supplemental crystalline methionine may be necessary to overcome this deficiency when high levels of CUP are included in broiler diets.

Dietary CUP supplementation linearly enhanced broiler immune and antioxidant indices. The observed increases in serum immunoglobulins (IgY, IgA, IgM) confirm the robust immunomodulatory properties of yeast cell walls. At the mechanistic level, mannans exposed on the outer layer of CUP cell walls act as pathogen-associated molecular patterns, which are recognized by pattern recognition receptors on gut-resident immune cells including macrophages and dendritic cells. This recognition triggers a non-infectious, feed-induced immune response that diverts metabolic energy and nutrients away from growth toward immune system maintenance, rather than serving a protective function against pathogens (Kiarie et al., 2022). This shift reflects a systemic low-grade inflammatory state rather than acute bacterial inflammation, manifesting as Th1 suppression, Th2-biased immune deviation, and chronic low-grade allergic reactions. This immune deviation is characterized by elevated IL-4 and IL-10 levels, which enhance humoral immunity while suppressing Th1-mediated pro-inflammatory cytokines such as IL-2 and TNF-α (Moore et al., 2001). As IL-2 is indispensable for IFN-γ synthesis (Schroder et al., 2004), the downregulation of IL-2 directly accounts for the reduced IFN-γ concentrations. Meanwhile, the increased activities of antioxidant enzymes (SOD, GSH-Px) together with reduced MDA levels further support the immunomodulatory and protective effects of CUP (Sheikh et al., 2020; Teng et al., 2023).

The cecal microbiota underwent a dose-dependent restructuring, with *Bacteroides* abundance increasing progressively with dietary inclusion levels. *Gemmiger* is primarily responsible for fiber degradation and short-chain fatty acid production. These taxonomic changes fundamentally altered the potential functions of the microbiome (Yin et al., 2023). This shift reflects an adaptive specialization toward degrading yeast glycans, facilitated by the extensive carbohydrate-active enzyme repertoire of *Bacteroides* (Lapébie et al., 2019). Although the increased Firmicutes-to-Bacteroidetes (F / B) ratio is traditionally associated with enhanced energy harvesting (Salaheen et al., 2017), its coincidence here with reduced growth suggests the microbiota was specialized for yeast polysaccharide fermentation rather than efficient energy extraction from the basal diet. Moreover, the enrichment of anaerobic taxa (e.g., *Anaeromassilibacillus*) and the dominance of supplemented yeast in the mycobiota underscore a multi-kingdom ecological shift. The proliferation of eukaryotic protists like *Blastocystis* in the CUP8 group warrants further study regarding its impact on gut homeostasis (Nash et al., 2017), indicating that high-level CUP supplementation significantly reshaped the cecal microbiota structure of broilers.

Overall, CUP is a valuable protein source in broiler nutrition. At 4% inclusion, it provides balanced protein nutrition and immunomodulatory benefits while supporting a favorable gut microbiota, resulting in optimal growth. At higher levels, its role shifts toward that of a bioactive immunostimulant and antioxidant, but this is offset by metabolic costs, reduced FI, impaired methionine digestibility, and profound microbial restructuring. These trade-offs establish 4% as the practical inclusion ceiling for balancing productivity and metabolic health. Some limitations should be noted. First, the metabolizable energy value of CUP was estimated from data for *Saccharomyces cerevisiae* in the Chinese Tables of Feed Composition and Nutritive Values; a dedicated assay is needed to confirm energy values. Second, the industrial-grade CUP used in this study had a CP content of 38%, a level lower than the 50% CP measured in laboratory-cultivated CUP. By contrast, the EE content of the industrial product reached 6%, which was higher than that of its laboratory-cultivated counterpart. The possible reasons for this result are the differences in the *C. utilis* culture medium and the unoptimized fermentation time. These factors should be considered when interpreting dose-response outcomes. Future research should focus on strategies such as methionine supplementation or synergistic use of cell-wall-degrading enzymes to mitigate nutritional constraints at high inclusion levels, and further elucidate the long-term consequences of CUP-induced microbial shifts.

## Conclusion

Under the experimental conditions of this study, quadratic regression analysis based on growth performance yielded a theoretically optimal dietary CUP inclusion range of 1.6% (based on d 1-21 BW) to 2.5% (based on d 1-21 FCR) for broilers. Nevertheless, supplementation with 4% CUP conferred additional functional benefits: it improved the apparent digestibility of dry matter, crude protein and amino acids, attenuated systemic inflammatory responses, and enhanced both humoral immunity and antioxidant capacity. Taking into account growth performance, immune-enhancing efficacy, and the marginal economic benefit of feed costs, a comprehensive recommended inclusion range of 1.6% to 4.0% is proposed for CUP in broiler diets over the entire 1-42 day rearing period. More specifically, an inclusion level of 2.5% is optimal for pursuing the best feed-to-gain ratio, while 4.0% is recommended to achieve maximum comprehensive production performance. For commercial flocks subjected to high-density breeding stress, the 2.5% inclusion level is advised to maintain intestinal microecological stability. For routine large-scale commercial production, a 4% dietary CUP supplementation level is recommended as the practical application standard.

## Ethics declaration

This study was conducted in accordance with the ARRIVE (Animal Research: Reporting of In Vivo Experiments) guidelines. This study was approved by the Laboratory Animal Welfare and Ethics Committee of the Institute of Animal Sciences, Chinese Academy of Agricultural Sciences. (Approval No. IAS2025-221).

## Author contributions

**Shuai Tan** designed and conducted the study, coordinated the animal trial, collected and analyzed the data, interpreted the results, and drafted the manuscript. **Jianshuang Ma** contributed to the experimental design, supervised the study, assisted with data analysis and interpretation, and critically revised the manuscript. **Xiaoyun Su, Xinxin Xu, Huoqing Huang, Tao Tu, Feifei Guan, Huiying Luo, Huimin Yu, Bin Yao** contributed to the conduct of the study, sample collection, data organization and interpretation, participated in the discussion of the results, and critically reviewed the manuscript. **Yuan Wang** conceived and supervised the project, contributed to the study design and interpretation of the findings, secured funding, and critically revised the manuscript. All authors contributed to the article, reviewed and approved the final version of the manuscript, and agreed to be accountable for all aspects of the work.

## Disclosures

We declare that we have no financial and personal relation-ships with other people or organizations that can in appropriately influence our work, and there is no professional or other personal interest of any nature or kind in any product, service and/or company that could be construed as influencing the content of this paper.

## Footnotes

Supplementary material associated with this article can be found, in the online version, at doi:10.1016/j.psj.2026.107749.
