# Supplementary material for: The single-cell protein from Candida utilis as a functional protein ingredient for broilers
Source: Poult Sci. 2026 Sep 11;105(12):107749. doi: 10.1016/j.psj.2026.107749 (PMC13634700; doi:10.1016/j.psj.2026.107749)
Supplement: Supplementary file 1 [file mmc1.docx]

**SUPPLEMENTAL MATERIALS**

**Supplementary Figure 1. Change in reducing sugars content measured at OD₅₄₀ₙₘ.**

**Supplementary Figure 2. Relative abundances of Firmicutes and Bacteroidetes in the cecal microbiota.**

| Items, % | CON | CUP4 | CUP8 | SEM^2^ | *P*-value |
| --- | --- | --- | --- | --- | --- |
| *Alistipes* | 10.87 | 8.47 | 6.97 | 1.17 | 0.426 |
| *Anaeromassilibacillus* | 0.579^b^ | 1.00^b^ | 3.11^a^ | 0.283 | <0.001 |
| *Bacteroides* | 2.17 | 1.41 | 3.65 | 0.477 | 0.193 |
| *Blautia* | 7.98^a^ | 6.42^ab^ | 4.01^b^ | 0.630 | 0.043 |
| *Candidatus_Enterousia* | 1.10 | 0.811 | 0.489 | 0.221 | 0.578 |
| *Clostridium* | 1.73 | 2.42 | 2.12 | 0.134 | 0.074 |
| *Eisenbergiella* | 1.67 | 0.924 | 1.53 | 0.186 | 0.198 |
| *Eubacterium* | 1.47 | 1.27 | 1.15 | 0.107 | 0.502 |
| *Faecalibacterium* | 1.45 | 0.733 | 1.51 | 0.264 | 0.412 |
| *Faecousia* | 1.31 | 1.28 | 2.86 | 0.381 | 0.211 |
| *Flavonifractor* | 1.14 | 1.15 | 0.820 | 0.200 | 0.789 |
| *Gemmiger* | 1.82 | 4.16 | 1.93 | 0.587 | 0.168 |
| *Lachnoclostridium* | 1.19 | 1.16 | 0.852 | 0.130 | 0.580 |
| *Lactobacillus* | 2.26 | 4.98 | 1.99 | 1.26 | 0.577 |
| *Ligilactobacillus* | 4.18 | 5.54 | 4.43 | 1.37 | 0.910 |
| *Mediterraneibacter* | 5.54 | 6.94 | 3.98 | 0.627 | 0.191 |
| *Pseudoflavonifractor* | 1.01 | 0.561 | 0.701 | 0.092 | 0.094 |
| *Ruminococcus* | 5.96 | 3.95 | 4.36 | 0.676 | 0.426 |
| *Streptococcus* | 3.23^a^ | 0.308^b^ | 0.532^b^ | 0.468 | 0.007 |
| *unclassified* | 0.420 | 0.211 | 0.253 | 0.041 | 0.069 |

**Supplementary Table 1. Species abundance of Function 01 in the species and functional contribution bar plot^1^.**

^1^CON was the control diet. The other 2 diets were supplemented with C. utilis protein at levels of 4% and 8%, and were named CUP4 and CUP8, respectively.

^3^ SEM, standard error of the mean.

^a-b^ Means with different superscripts in the same row are significantly difference.

**Supplementary Table 2. Species abundance of Function 02 in the species and functional contribution bar plot^1^.**

| Items, % | CON | CUP4 | CUP8 | SEM^2^ | *P*-value |
| --- | --- | --- | --- | --- | --- |
| *Alistipes* | 10.4 | 8.08 | 7.14 | 1.12 | 0.498 |
| *Anaeromassilibacillus* | 0.504^b^ | 0.887^b^ | 2.79^a^ | 0.252 | <0.001 |
| *Bacteroides* | 2.55 | 1.55 | 4.10 | 0.491 | 0.132 |
| *Blautia* | 8.67^a^ | 7.30^ab^ | 4.44^b^ | 0.660 | 0.036 |
| *Candidatus_Enterousia* | 1.97 | 1.42 | 0.869 | 0.388 | 0.560 |
| *Clostridium* | 1.97^b^ | 2.75^a^ | 2.45^ab^ | 0.138 | 0.040 |
| *Eisenbergiella* | 1.76 | 0.971 | 1.62 | 0.185 | 0.153 |
| *Eubacterium* | 1.55 | 1.34 | 1.18 | 0.102 | 0.363 |
| *Faecalibacterium* | 1.31 | 0.634 | 1.36 | 0.230 | 0.357 |
| *Faecousia* | 1.42 | 1.38 | 2.99 | 0.371 | 0.180 |
| *Flavonifractor* | 1.55 | 1.63 | 1.31 | 0.222 | 0.863 |
| *Gemmiger* | 1.75 | 3.32 | 1.70 | 0.423 | 0.198 |
| *Lachnoclostridium* | 1.39 | 1.32 | 0.976 | 0.127 | 0.444 |
| *Lactobacillus* | 1.98 | 4.81 | 1.70 | 1.22 | 0.526 |
| *Ligilactobacillus* | 3.43 | 4.61 | 3.55 | 1.19 | 0.907 |
| *Mediterraneibacter* | 5.74 | 6.62 | 3.84 | 0.564 | 0.160 |
| *Pseudoflavonifractor* | 1.35 | 0.875 | 1.00 | 0.111 | 0.170 |
| *Ruminococcus* | 4.88 | 3.30 | 3.60 | 0.546 | 0.442 |
| *Streptococcus* | 2.65^a^ | 0.258^b^ | 0.450^b^ | 0.380 | 0.006 |
| *unclassified* | 0.722^ab^ | 0.611^b^ | 0.871^a^ | 0.034 | 0.006 |

^1^CON was the control diet. The other 2 diets were supplemented with C. utilis protein at levels of 4% and 8%, and were named CUP4 and CUP8, respectively.

^3^ SEM, standard error of the mean.

^a-b^ Means with different superscripts in the same row are significantly difference.
